# Movement of feeder-using songbirds: the influence of urban features

**DOI:** 10.1038/srep37669

**Published:** 2016-11-23

**Authors:** Daniel T. C. Cox, Richard Inger, Steven Hancock, Karen Anderson, Kevin J. Gaston

**Affiliations:** 1 Environment & Sustainability Institute, University of Exeter, Penryn, Cornwall TR10 9EZ, U.K; 2 Global Ecology Lab, University of Maryland, Maryland, MD 20742, U.S

## Abstract

Private gardens provide vital opportunities for people to interact with nature. The most popular form of interaction is through garden bird feeding. Understanding how landscape features and seasons determine patterns of movement of feeder-using songbirds is key to maximising the well-being benefits they provide. To determine these patterns we established three networks of automated data loggers along a gradient of greenspace fragmentation. Over a 12-month period we tracked 452 tagged blue tits *Cyantistes caeruleus* and great tits *Parus major* moving between feeder pairs 9,848 times, to address two questions: (i) Do urban features within different forms, and season, influence structural (presence-absence of connections between feeders by birds) and functional (frequency of these connections) connectivity? (ii) Are there general patterns of structural and functional connectivity across forms? Vegetation cover increased connectivity in all three networks, whereas the presence of road gaps negatively affected functional but not structural connectivity. Across networks structural connectivity was lowest in the summer when birds maintain breeding territories, however patterns of functional connectivity appeared to vary with habitat fragmentation. Using empirical data this study shows how key urban features and season influence movement of feeder-using songbirds, and we provide evidence that this is related to greenspace fragmentation.

As urbanization increases globally, green spaces in cities and towns are becoming of greater importance for the provision of ecosystem services^1^,^2^. Domestic gardens are a major component of these green spaces^3^,^4^,^5^. They constitute easily accessible and immediate locations where people can interact with nature, enabling access to the broad range of health and well-being benefits that nature provides^6^,^7^. Birds are a key component of garden wildlife^8^ and for many people their interactions with wild birds may form the main daily wildlife interaction^9^. Watching birds and listening to their song have been shown positively to influence human psychological well-being^10^,^11^,^12^,^13^,^14^,^15^. Given these benefits, it is perhaps unsurprising that the provision of supplementary food is the most popular form of wildlife gardening (reviewed by ref. 11).

Domestic green spaces are often characterised by numerous small and densely packed gardens that are utilised and managed by an equivalent number of households^4^,^5^. This results in individual birds typically moving between multiple gardens to forage and visit feeders, where they will be seen by, and so provide benefits to, multiple people. The ability of birds to move between gardens thus increases the potential benefits that any individual bird can provide, with actual movement being determined by the structures of the gardens themselves, including their geographical location in relation to one another, the habitat for birds within the gardens, and the surrounding urban features.

Previous studies in urban areas have estimated connectivity for birds within and between public green spaces^16^,^17^,^18^,^19^,^20^. These studies suggest that vegetation between green spaces preserves connectivity, while multiple barriers, such as roads and rivers, cumulatively decrease landscape permeability. However, despite the clear importance of domestic gardens in generating connectivity in their own right and for facilitating connectivity between larger green spaces^1^,^4^, fragmented land ownership and management mean that their role in shaping connectivity is largely unexplored empirically^21^,^22^. Indeed, how structural patterns of key features that distinguish different urban forms affect the flow of birds around the landscape is currently unknown. In the wider landscape there is seasonal variation in connectivity that is related to habitat quality and quantity^23^,^24^, therefore we might expect this also to occur across domestic gardens and to vary across different urban forms. For example, birds defend smaller home ranges when breeding in summer than when foraging more widely in winter.

Birds that utilize feeders provide an ideal group for exploring the relationships among urban form, connectivity and cultural service delivery. Radio Frequency Identification (RFID) technology provides a means of doing so. This can produce a continuous record of the time and date of when an individual bird carrying a Passive Integrated Transponder (PIT) tag visits a resource patch. Networks of RFID readers can thus be used to record individual movement in time and space as birds visit different feeders. This allows the influence of different urban features on structural and functional connectivity to be determined, structural connectivity here being the presence-absence of connections made by birds moving between feeders and functional connectivity the frequency of those connections. We set up three networks of custom-designed low-powered RFID equipped bird feeders within domestic gardens in the Cranfield triangle in Southern England, UK, with each network within a different urban form that is common in Europe; these had, respectively, low, medium or high green space fragmentation. We used hyperspectral and LiDAR data to characterise the landscape structure through which tagged birds were likely to move between feeder pairs within each network. There were 17 feeders per network, and these were operated continuously over a 12-month period to explore two general questions:

1. How do different features within each urban form, and season, influence general patterns of structural and functional connectivity for birds?
2. Are there general patterns of structural and functional connectivity across these forms?

## Results

**Figure 1:**
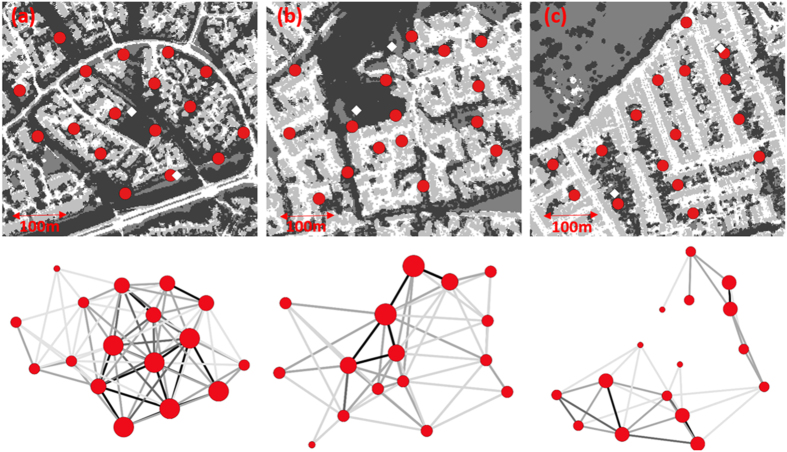
The frequency of connections (i.e. functional connectivity) of two species of garden bird moving between bird feeders, within (**a**) the network of low fragmentation, (**b**) the network of medium fragmentation, (**c**) the network of high fragmentation. Connections occurred over a 12-month period. The upper panel rasters were generated using hyperspectral and LiDAR data (Appendix S1), we show the location of rfid bird feeders in red. Habitat classification: white; vegetation free surfaces at ground level, light grey, buildings; medium grey, grass & low lying vegetation, dark grey; vegetation (at 2 m resolution). The lower panels show the frequency of each connections (black line, >100; dark grey line, >50–100; medium grey line, >10–50; light grey line, 1–10) and the total number of connections made by each feeder (divided into 4 categories denoted by increasing size and brightness of the red circle: 0; 10; 50; 100; >200). ♦ Bird catching locations. Images were created in R version 3.1.2^34^. *To increase the clarity of the image only those connections that occurred between feeder pairs that were less than the mean distance between all feeder pairs are shown (<213 m); this only loses 9% of the total connections made, and does not exclude any feeder pairs with =>10 connection.

In total we tagged 452 individuals of two common species of feeder-using birds (blue tit *Cyanistes caeruleus* and great tit *Parus major*) between June 2013 and August 2014 (see Supplementary Table S1 and Fig. S1). We divided the year into four equal seasons: summer, autumn, winter, spring. We then considered that a tagged bird visiting first one and then a different feeder within each network and within each season made a connection, with data collection starting on the 1^st^ September 2013. Across the three networks, 51% (±2 SD) of tagged individuals made one or more connections between feeders (n = 9,848; Fig. 1). Eighty-eight percent of connections occurred within two days (n = 8,652; See Supplementary Fig. S2). We discarded from the analysis connections that took longer to make because we considered there was a high probability that birds travelled to the second feeder via a non-direct route. Using hyperspectral and LiDAR data we categorised the habitat in an ellipsoid between feeder pairs to establish variation in urban form across the three networks of RFID readers (Table 1). For each feeder pair we calculated the distance between feeders, the shortest distance between feeders and a bird catching site, and finally within each ellipsoid we calculated vegetation cover and the number of road gaps (Table 1; Fig. 1).

### Urban Features Within Forms and Season

The first stage of our analysis tested for the effect of different urban features and season on structural connectivity (the presence or absence of a connection between feeder pairs in any season) and functional connectivity (the frequency of these movements between feeder pairs in any season) within each of the networks.

**Figure 2:**
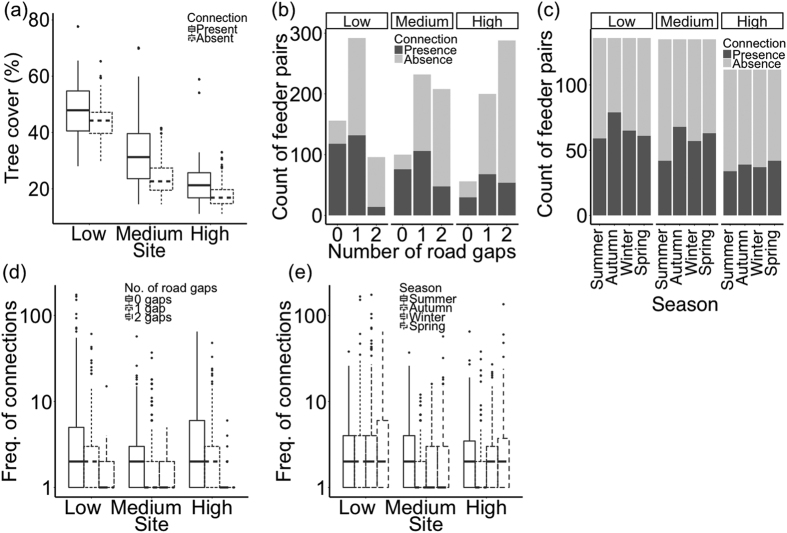
The effect of urban features, and season, on structural (**a–c**) and functional (**d,e**) connectivity across networks, for feeder-using birds. Structural connectivity (presence-absence of connections): (**a**) the percentage vegetation cover of feeder pairs with connections present and absent by network; (**b**) the number of feeder pairs against the number of road gaps and (**c**) the number of feeder pairs that formed connections in each season. Functional connectivity (frequency of connections where ≥1 connection was made (log 10 on y-axis): (**d**) frequency against the number of road gaps by network, and (**e**) frequency against season.

For structural connectivity, in each network the likelihood of a connection being present between feeders (i.e. connectivity) increased with the percentage vegetation cover (Table 2a; Fig. 2a), while the presence of one or more road gaps did not affect movement (Table 2a; Fig. 2b). In the networks of low and medium fragmentation, connectivity decreased with distance between feeders. In the network of low fragmentation, connectivity was lowest in summer and highest in the autumn and winter (Table 2a; Fig. 2c). In the network of medium fragmentation, connectivity was higher across the year relative to summer (Table 2a; Fig. 2c). In the network of high fragmentation, connectivity was highest in spring relative to the other seasons (Table 2; Fig. 2c). Great tits moved between fewer feeder pairs than blue tits in the medium and high fragmentation networks, while there was decreased movement with increasing distance from the ringing site in the network of medium fragmentation (Table 2).

For functional connectivity, vegetation cover increased the frequency of movement across all networks (Table 2b). In the network of low and medium fragmentation the frequency of connections decreased with increasing distance between feeders, while decreasing in all networks in the presence of road gaps (Table 2b; Fig. 2d). There was seasonal variation in connectivity relative to summer that varied across networks; in the network of low fragmentation connectivity was higher across the year relative to summer, while in the network of medium fragmentation connectivity was lowest in autumn and winter (Table 2b; Fig. 2e). In the network of low fragmentation connectivity was lowest in autumn and highest in spring (Table 2b; Fig. 2e). Movement decreased with distance to ringing site in the network of medium fragmentation (Table 2b).

### Patterns of Movement Across Urban Forms

**Figure 3:**
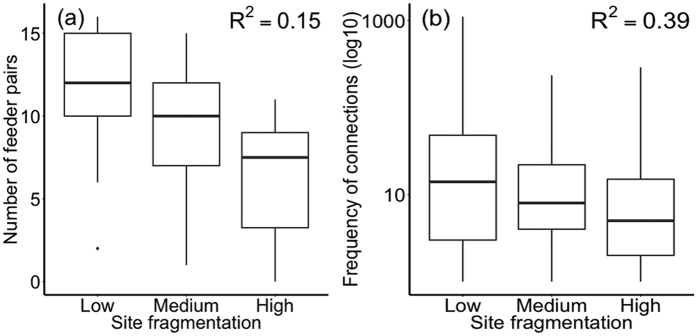
Comparison of the movements of tagged birds between feeder pairs across networks: (**a**) Structural connectivity (the numbers of feeders that each feeder is connected to), and (**b**) Functional connectivity (the total number of connections made to each feeder). Pseudo R^2^ from quasi-models shown.

The second stage of our analysis explored general patterns of structural and functional connectivity across the three networks. We found that structural connectivity decreased significantly across the three networks with increasing green space fragmentation (low fragmentation, 77%; medium fragmentation, 68%; high fragmentation, 55% of feeder pairs had connections; ANOVA of quasi-binomial model, network *Χ*^2^ = 20.4, df = 2, *P* = <0.0001; R^2^ = 0.15; Table 3a; Fig. 3a). Functional connectivity was greatest in the network of low fragmentation, whilst there was no difference between the networks of medium and high fragmentation (ANOVA of quasi-Poisson model, network *Χ*^2^ = 1708.0, df = 2, *P* = <0.0001; R^2^ = 0.39; Table 3b; Fig. 3b). In both models connectivity decreased with distance between feeder pairs (Table 3).

## Discussion

Understanding how the spatial and temporal heterogeneity of urban green spaces determines patterns of connectivity is critical for manipulating the flow of ecosystem services around where people live and work. This is the first study that uses empirical data to model both structural (a measure of the ability of birds to move through the landscape) and functional (a measure of the frequency of movement of individuals) connectivity. Due to the labour and time intensive nature of a study of this kind it is not possible within realistic budgets to test variation in movement across large numbers of networks (e.g. replicating different urban forms or across a gradient of forms). However, we show that key urban features and season influenced movement within three distinct urban forms, and (recognising the limitations of a three-site comparison) there is evidence that overall levels of connectivity varied across forms with increased movement being associated with reduced green space fragmentation.

In the network of low fragmentation movement focused around a large central cluster with birds using green corridors to move to, and between, feeders away from ringing sites, and movement then decreased as green spaces became more fragmented (Fig. 1a). This was supported in our models with both forms of connectivity increasing with vegetation cover, and decreasing with distance between feeder pairs. Functional but not structural connectivity was negatively correlated with the presence of road gaps, suggesting that birds do travel between green fragments but roads cause resistance to frequent movement. Connectivity varied by season, being lowest in the summer probably as a consequence of breeding season territoriality. Structural connectivity was then greatest in autumn, at a time when birds are engaged in natal and post-breeding dispersal, before decreasing in winter as garden songbirds become more settled in their wintering territories^25^. Relative to the summer, functional connectivity increased during the year, peaking in winter when food supplies were constrained and birds moved frequently between known feeders. The increased availability of vegetation and green corridors in this network may allow ecological processes most closely to mimic those we expect to see in more natural habitats^25^,^26^.

In the network of medium fragmentation movement mostly occurred within the central woodland area and along vegetation corridors that largely originated from the wooded area, while there was little movement between feeders within suburban gardens (Fig. 1b). Connectivity increased with vegetation cover, but decreased with distance from ringing site indicating that suburban gardens reduced landscape permeability. Functional connectivity but not structural connectivity decreased in the presence of road gaps supporting this conclusion. Structural connectivity decreased in summer when birds remain in their breeding territories, but was higher across the rest of the year possibly because birds were more likely to explore into gardens. Functional connectivity decreased in autumn and winter relative to spring and summer, suggesting that birds visited feeders opportunistically as they passed through a garden as opposed to having established wintering territories^27^. Great tits showed lower structural connectivity relative to blue tits in the networks of medium and high fragmentation, possibly because they were less abundant around these networks (see Supplementary Table S1). Great tits tend to forage in mature trees and so may have been more reluctant to move to feeders away from vegetated corridors possibly as a consequence of relatively high blue tit populations^28^.

In the network of high fragmentation, green space corridors between streets with terraced housing appeared to funnel movement (Fig. 1c). This could in part be a consequence of the ringing locations being located at the periphery of the network, however, there was very little movement into the relatively vegetation-impoverished central green islands located between the two ringing locations suggesting that crossing terraced streets causes resistance to movement relative to moving along green spaces. In this highly fragmented urban form structural and functional connectivity were positively correlated with vegetation but unaffected by distance between feeder pairs (at the scale of the study). Again, functional but not structural connectivity was negatively correlated with the presence of road gaps. The presence and frequency of connections were greatest in spring, possibly because birds were searching for breeding territories^29^. At a time when birds are undergoing post breeding dispersal connectivity was least in autumn, suggesting that tagged birds did not linger but instead were passing through.

There was variation in structural connectivity across networks, with 22% more feeder pairs forming connections in the network of low compared to that of high fragmentation (Fig. 3a). Despite the large numbers of possible drivers behind the movement of feeder-using birds (such as non-experimental bird feeding, density of cat populations or intra-specific territoriality) this study shows a gradient in the ability of birds to move between different feeders with green space fragmentation. Of particular concern is that over the whole 12-month period tagged birds failed to visit feeders within the relatively impoverished green fragments in the highly fragmented network, suggesting that residents in these areas will effectively be cut off from this form of nature connection. High quality green space maintains the flow of birds across a broad range of gardens, providing opportunities to a greater number of households to interact with birds.

Functional connectivity was also greatest in the network of low fragmentation. However, we found no difference in this regard between the networks of medium and high fragmentation, and their relatively low functional connectivity suggests that the associated gardens contained relatively poor quality habitat so that birds either passed through the networks, or the networks were too fragmented to establish territories. The networks of medium and low fragmentation both contained green corridors surrounding residential houses, mainly differing in the relative absence of trees in the medium fragmented network. Large trees are known to be keystone structures in urban areas for increasing connectivity^30^. Their loss may be a key factor in contributing towards the reduced movement into gardens in this network. This is of concern because this urban form is representative of new developments (within the last 25 years) that are common in European cities.

The phenomenon of garden bird feeding is growing in many developed regions, with bird feeders now being a common component of urban areas^11^. The provision of large quantities of supplementary food drives movement patterns and abundances of feeder-using birds^31^,^32^. Understanding how individual birds move between gardens with bird feeders, thus provides real world insights into actual movement in the urban landscape. Watching birds at garden feeders provides people with a sense of increased psychological well-being, feelings of relaxation^13^ and of being connected to the natural world^12^,^13^. Listening to bird song and watching birds in the garden have been shown to contribute towards attention restoration and recovery from stress^10^,^15^. Greater connectivity will increase the probability that birds will be seen by and so provide pleasure to multiple households, thus multiplying the benefits provided by any individual bird even though it is usually impossible for households to distinguish between these individuals.

Understanding and quantifying the relationship between the movement of wildlife and urban features is key for ecologically sensitive planning to aid the flow of cultural services within existing cities. Given that many existing urban areas have relatively inflexible urban forms, improved movement could be achieved through targeted greening at focal points of connectivity (e.g. in specific parks and gardens). The applied use of high quality remote sensing techniques, landscape ecology principles and theory (e.g. patch and matrix frameworks) and systematic conservation planning approaches to identify and exploit these focal points has the potential disproportionately to increase the movement of birds into impoverished areas. Targeted greening could be achieved through a combination of management by local authorities, while also raising public awareness of the importance of best practise habitat management in their own gardens. Future research needs to focus on producing real world tools for ease of use by public and private stakeholders for mapping connectivity and identifying and exploiting these focal points. How we improve existing forms and design new residential areas will have a large impact on the daily nature exposure of the people that live there, and thus has the potential to mitigate many of the negative impacts of urbanisation.

## Materials and Methods

### Study Networks

The focal geographical area for this study is what has been termed the ‘Cranfield triangle’ (52°07′N, 0°61′W). Located ~60 km to the north of London, UK, the main urban areas consisted of Milton Keynes, Luton and Bedford, having a combined population of c. 524,000. Each study network occupied approximately 0.5 km^2^, with its precise location determined by the presence of suitable areas in which to mist-net feeder-using birds. Relative to each other, the networks consisted of one characterized by physically well connected green spaces with high levels of vegetation cover and detached houses (in Milton Keynes; Network of low fragmentation; Fig. 1a), a network with intermediate levels of connected green spaces and vegetation cover with semi-detached houses (in Luton; Network of medium fragmentation; Fig. 1b), and a network with terraced houses consisting of fragmented green spaces with low levels of vegetation cover (in Bedford; Network of high fragmentation; Fig. 1c).

### Experimental Design

Bird feeders with integrated RFID reader and antenna were constructed utilising custom-made Arduino components (Relchron LTD, Kirkcaldy, Scotland; ref. 33). Rectangular antennas (40 mm × 32 mm) recording at 125 kHz were fixed using cable ties and epoxy plastic to the underside of a single perch on a standard medium sized seed feeder (360 mm, The Royal Society for the Protection of Birds, Sandy, UK). Other perches on the feeder were removed and associated feeding ports sealed closed to ensure all visitations were recorded. Data-loggers were programmed to alternate 400 ms of recording with 400 ms of pause. When a PIT tag was within range of the antenna the data logger recorded the time and date along with the tag’s unique identification number onto a 4 gb memory card (SanDisk, Milpitas, USA). The readers were powered 24 hours a day by a 12 v battery (Xplorer 88 amp deep cycling, Alpha-batteries, Rochdale, UK), allowing continuous monitoring of feeder usage.

Each network considered of 17 RFID bird-feeding stations. Each station was set up in a private garden averaging 81 m (±20 m SD) from its nearest neighbour station. Within the constraint of there being suitable locations within gardens, feeders were placed ~0.5 m from cover for birds, although the actual position was inevitably influenced by property residents who volunteered use of their gardens (Fig. 1). Feeders were installed in gardens up to six weeks before the experiment began to allow birds to familiarise with them. Every feeding station consisted of a bird feeder with an RFID reader, antenna and power source, a bird feeder stand (Kingfisher, Paddington, UK) and a universal squirrel baffle (Gardman, Peterborough, UK). The bottom of the feeder was suspended ~1 m above the ground. We used the same seed mix in all feeders throughout the experiment (Summer Seed Mix, Haithes, Bird Food specialists, UK). The feeder was maintained by the property residents to ensure that birds could access seed at all times, and a researcher visited all stations every 30 days to replace the battery and to download the data. All 51 feeding stations were fully operational between 1^st^ September 2013 and 31^st^ August 2014.

### Tagging in the Field

Birds were caught and tagged in private green spaces at two locations in each network, chosen to maximize catching rates and with the nearest RFID feeding station approximately 15 m from the closest net (Fig. 1). Mist-nets and tape lures were used to catch two garden species that commonly visit bird feeders: blue tit *Cyanistes caeruleus* and great tit *Parus major*. Mist netting was carried out intensively during the experimental set-up period and then monthly in each network, with birds being fitted with British Trust for Ornithology (BTO) metal rings and with a PIT tag, which was fully moulded into an 8 mm plastic ring (IB Technology, Aylesbury, U.K.). All bird ringing was carried out under BTO license A/5780 with a special endorsement to attach PIT tags to target species. This research was conducted with approval from, and in accordance with, the University of Exeter Biosciences ethical review committee, project number 2013/72.

### Landscape Characterization

We characterized the spatial pattern of vegetation and non-vegetation, and canopy height in the landscape for each network using airborne remote sensing, specifically with hyperspectral and LiDAR data (see Supplementary Appendix S1). We described the habitat that birds are likely to use when moving between any pair of feeders within a network by applying a buffer around and between each pair of feeders equal to 0.25 times the distance between them. Based on previous studies, such an ellipsoid, with a constant length to width ratio of three, represents a reasonable area for a bird seeking to move between feeders (for example, see Supplementary Fig. S3; see also refs 19 and 34). Within each buffer we calculated the percentage of pixels containing tall vegetation (>0.7 m; vegetation cover according to the pixel values exceeding a basic threshold for a vegetation index; Table 1; see Supplementary Appendix S1). Finally, we counted the number of road gaps between each feeder pair, where a road gap was considered to be present when a road dissected the buffer (for example; see Supplementary Fig. S3). At the spatial extent of study, three or more road gaps were rare and so these were pooled with two road gaps. This was then treated as a three-level factor of 0–2 road gaps (Table 1). We calculated the distance in meters of the closest feeder of each pair with the closest ringing site (termed here as ringing site distance; mean = 42 m, SD = 46 m). We then divided the year into four equal seasons: autumn (1^st^ Sep–30^th^ Nov), winter (1^st^ Dec–28^th^ Feb), spring (1^st^ Mar–31^st^ May) and summer (1^st^ Jun–30^th^ Aug 2014). To assess the probability of whether a bird passed within the ellipsoid between feeders, we calculated the time taken to make each connection.

### Statistical Analysis

#### Urban Features within Forms and Season

All statistical analyses were performed in R version 3.1.2^35^, with mixed effects models built using the lme4 package^36^. To explore how different features within the three networks determined seasonal movement of feeder-using birds we used a hurdle modelling framework^37^. The hurdle model consisted of a binomial model (presence-absence of at least one connection in any season by species as the response variable, with one replication per species, per feeder pair, per season) and a count effect model (frequency of these connections in any season as the response variable, with one replication per individual tagged bird, per feeder pair where connections ≥1) based on a Poisson distribution truncated at 0 (i.e. no stochastic absence of connection). A hurdle modelling approach differentiated the effects of network on structural and on functional connectivity, and better accommodated marked overdispersion in our response variable than using a Poisson model^37^.

To characterise the role of urban structures on movement in each network we then built binomial and Poisson mixed effect models for each network separately. We standardized the continuous variables vegetation cover, distance between feeder pair and ringing site distance (i.e. each was rescaled to have a mean of zero and a standard deviation of 1). To explore structural connectivity we built a generalized linear mixed model (GLMM) with a binomial error distribution to test for the effect of vegetation cover, distance between feeder pair, number of road gaps and season on the presence-absence of connections between feeder pairs (see Table 1 for a summary of covariates by network). We also included species and the distance from ringing sites as covariates. Finally, to control for replication of feeder pairs across the seasons, for each feeder station we included a unique identification number (FeederID) within network as two random effects. To explore functional connectivity we built a GLMM with a Poisson error distribution to test the effect of the same predictor variables on the frequency of connections between feeders at the individual level (i.e. where connections ≥ 1). We included three random effects; FeederID for each feeder station to control for replication of feeder pairs across the season and a unique tag number to control for multiple individuals moving between the same feeder pair in any season.

#### Patterns of Movement Across Urban Forms

To explore general patterns of movement across the networks, we pooled connections by season before again using a hurdle modelling framework. The hurdle model consisted of a quasi-binomial model (presence/absence of at least one connection between feeder pairs, with one replication per feeder pair) and a count effect model (frequency of connections, with one replication per feeder pair where connections ≥1). It was assumed that general patterns of movement and associated cultural service provision by feeder-using songbirds were dependent on individual birds and not species, so we pooled connections by species. In each case we then tested for the effect of network type (included as a three level factor: low fragmentation; medium fragmentation; high fragmentation) on the response variable. We included distance between feeder pairs within feeder groups (in meters) as a covariate to control for slight variation between networks (Table 1).

## Additional Information

**How to cite this article**: Cox, D. T. C. *et al*. Movement of feeder-using songbirds: the influence of urban features. *Sci. Rep.* **6**, 37669; doi: 10.1038/srep37669 (2016).

**Publisher’s note:** Springer Nature remains neutral with regard to jurisdictional claims in published maps and institutional affiliations.

## Supplementary Material

Supplementary Material

## Figures and Tables

**Table 1:** Summary of urban features per feeder pair in each of the three networks: mean distance between pairs of feeders, mean vegetation cover within the buffer and the total number of road gaps crossing buffers (as a measure of overall green space fragmentation).

Network	Distance between feeders (metres)	% Vegetation cover	Total number of road gaps
Low fragmentation	203 (±92)	45.8 (±8.4)	121
Medium fragmentation	218 (±98)	28.1 (±10.4)	182
High fragmentation	213 (±98)	19.3 (±7.6)	302

Associated standard errors are shown in brackets.

**Table 2:** The relationships between (a) structural (binomial) and (b) functional (Poisson) connectivity and the presence of key urban features and season by network, for two feeder-using songbirds.

	Low fragmentation		Medium fragmentation		High fragmentation	
	Estimate (±se)	CI (2.5%; 97.5%)	Estimate (±se)	CI (2.5%; 97.5%)	Estimate (±se)	CI (2.5%; 97.5%)
a) Structural connectivity						
Intercept	−1.7 (±0.4)***	−2.7; −0.7	−1.8 (±0.4)***	−2.6; −0.8	−2.4 (±0.6)***	−3.7; −1.0
Vegetation cover	0.3 (±0.1)**	0.1; 0.6	0.8 (±0.2)***	0.4; 1.1	0.5 (±0.2)**	0.1; 0.9
Distance	−1.3 (±0.1)***	−1.6; −1.0	−1.2 (±0.2)***	−1.6; −0.9	−0.2 (±0.3)	−0.7; 0.4
1 road gap	−0.1 (±0.3)	−0.6; 0.4	−0.3 (±0.3)	−0.8; 0.3	0.1 (±0.4)	−0.8; 0.9
2 road gaps	−0.2 (±0.4)	−1.0; 0.7	−0.0 (±0.4)	−0.7; 0.7	−0.7 (±0.6)	−1.8; 0.4
Autumn	1.5 (±0.2)***	1.0; 2.0	0.8 (±0.3)**	0.3; 1.4	0.3 (±0.3)	−0.4; 1.0
Winter	0.8 (±0.2)**	0.3; 1.3	0.6 (±0.3)*	0.0; 1.1	0.6 (±0.3)	−0.1; 1.2
Spring	0.1 (±0.2)	−0.4; 0.6	0.8 (±0.3)**	0.3; 1.3	0.9 (±0.3)**	0.3; 1.6
Species	0.0 (±0.2)	−0.3; 0.4	−1.1 (±0.2)***	−1.4; −0.7	−1.6 (±0.2)***	−2.1; −1.1
Ring site distance	−0.2 (±0.3)	−0.7; 0.2	−0.5 (±0.2)**	−0.8; −0.1	−0.3 (±0.3)	−0.8; 0.4
R^2^_GLMM(*m*)_		0.29		0.4		0.28
R^2^_GLMM(*c*)_		0.57		0.55		0.62
b) Functional connectivity						
Intercept	1.2 (±0.3)***	0.6; 1.7	1.3 (±0.2)***	1.0; 1.6	1.2 (±0.3)***	0.5; 1.7
Vegetation cover	0.1 (±0.0)*	0.0; 0.2	0.2 (±0.1)**	0.1; 0.3	−0.2 (±0.1)*	−0.5; −0.1
Distance	−0.5 (±0.0)***	−0.6; −0.5	−0.3 (±0.1)***	−0.4; −0.1	−0.2 (±0.1)	−0.5; 0.0
1 road gap	−0.4 (±0.1)***	−0.6; −0.3	−0.2 (±0.1)**	−0.4; −0.1	−0.1 (±0.2)***	−1.5; −0.9
2 road gaps	0.3 (±0.2)	−0.1; 0.7	−0.3 (±0.2)*	−0.6; −0.0	−2.4 (±0.4)***	−2.9; −1.4
Autumn	0.3 (±0.1)***	0.1; 0.4	−0.5 (±0.1)***	−0.7; −0.4	−0.3 (±0.1)**	−0.5; −0.1
Winter	0.4 (±0.1)***	0.3; 0.6	−0.5 (±0.1)***	−0.7; −0.4	−0.0 (±0.1)	−0.2; 0.2
Spring	0.2 (±0.1)**	0.1; 0.4	0.1 (±0.1)	−0.1; 0.3	0.6 (±0.1)***	0.4; 0.8
Species	0.2 (±0.2)	−0.1; 0.5	−0.1 (±0.1)	−0.3; 0.1	−0.0 (±0.2)	−0.5; 0.4
Ring site distance	−0.1 (±0.1)	−0.2; 0.0	−0.2 (±0.1)**	−0.3; −0.0	−0.1 (±0.2)	−0.4; 0.2
R^2^_GLMM(*m*)_		0.13		0.15		0.17
R^2^_GLMM(*c*)_		0.22		0.17		0.23

We show parameter estimates with standard errors and confidence intervals (CI) for factor levels relative to a comparative base factor level (0 road gaps, summer and blue tits, respectively). Significant variables and factor levels are shown as: *P < 0.05; **P < 0.01; ***P < 0.001. We show the marginal R^2^_GLMM(*m*)_ and conditional R^2^_GLMM(*c*)_.

**Table 3:** Hurdle model testing for the relationships between networks on overall levels of movement of feeder-using garden bird: (a) Structural connectivity; (b) Functional connectivity.

	Estimate (±se)	t value	CI (2.5%; 97.5%)
*a*) *Structural connectivity*			*pR*^*2*^ = *0.15*
Intercept	1.7 (±0.3)	5.6***	1.2; 2.4
Medium fragmentation	1.0 (±0.3)	3.7***	0.5; 1.6
Low fragmentation	1.1 (±0.3)	4.1***	0.6; 1.7
Distance	−0.008 (±0.001)	−6.9***	−0.01; −0.006
*b*) *Functional connectivity*			*pR*^*2*^ = *0.39*
Intercept	5.0 (±0.2)	20.2***	4.9; 6.0
Medium fragmentation	0.02 (±0.3)	0.1	−0.6; 0.5
Low fragmentation	0.6 (±0.2)	2.4*	0.2; 1.1
Distance	−0.1 (±0.001)	−7.5***	−0.02; −0.01

We show parameter estimates and associated standard errors, t values and confidence intervals (CI) for medium and low fragmentation networks relative to the base factor level of the high fragmentation network. Significant factor levels are shown as: *P < 0.05; **P < 0.01; ***P < 0.001. The pseudo R^2^ is McFaddens.
