# Supplementary material for: Movement of feeder-using songbirds: the influence of urban features
Source: Sci Rep. 2016 Nov 23;6:37669. doi: 10.1038/srep37669 (PMC5120271; doi:10.1038/srep37669)
Supplement: Supplementary Material [file srep37669-s1.pdf]

Supplementary Information

Movement of feeder-using songbirds: the influence of urban features

Daniel T. C. Cox*, Richard Inger, Steven Hancock, Karen Anderson & Kevin J. Gaston

Environment & Sustainability Institute, University of Exeter, Penryn, Cornwall TR10 9EZ, U.K.

Corresponding author. E-mail: dan.t.cox@googlemail.com

Appendix S1. Characterization of the urban form at each site.

Table S1. The ringing effort and number of tagged birds for each site.

Figure S1. Seasonal rates of tagging birds.

Figure S2. Time taken to form individual connections.

Figure S3. Examples of determination of habitat characteristics between feeder pairs.

Appendix S1. Characterization of the urban form at each site. The urban form of each site was characterized using airborne hyperspectral (Eagle spectrometer) and LiDAR (Leica ALS50-II) data collected by the Natural Environment Research Council (NERC) Airborne Research and Survey Facility (ARSF) aircraft in July and September 2012. The normalized difference vegetation index (NDVI;[1]) was calculated from the hyperspectral data using a red band focused at 570 nm and a near infra-red band centred at 860 nm with a spatial resolution of 2 m. Histograms of NDVI were examined and a threshold of 0.2 identified as being suitable to separate vegetated (NDVI>=0.2) from non-vegetated (NDVI<0.2) pixels[2]. The LiDAR data were used in discrete return mode, with up to four returns per laser pulse. The laser point density was between one point per 25cm^2 and one point per 2 m^2, depending on flight line overlap. The lastools software (http://rapidlasso.com/lastools/) 'lasground' function was used to find ground returns within the LiDAR point cloud. Pixels (2 m resolution) with an NDVI greater than 0.2 and a mean height of first return more than 0.7 m above the ground were marked as tall vegetation; this was defined as suitable habitat for birds[3]. Heights from discrete return LiDAR are well-known to produce biased results over vegetation[4] and so this 0.7 m threshold may have represented a more variable vegetation threshold height, and since that bias is most usually an underestimation, it could correspond to taller vegetation (up to 1.7 m tall).

Table S1: The ringing effort and number of tagged birds for each site.

Site	Days	Net meter hours	Blue tit	Great tit	Total bird's
Low fragmentation	31	2710	70	100	170
Medium fragmentation	21	2496	105	65	170
High fragmentation	36	2520	82	30	112
**Total**	**88**	**7726**	**257**	**195**	**452**

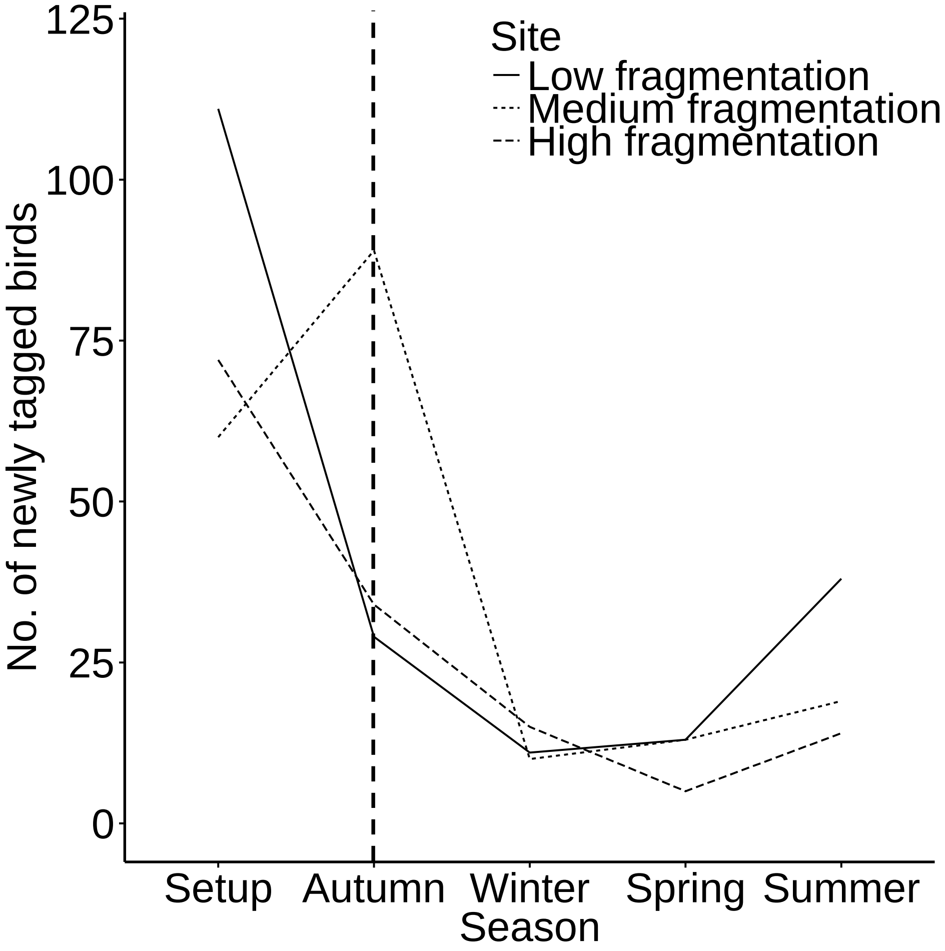

Fig. S1. Seasonal rates of tagging birds. The number of newly tagged individuals of two common garden species by season and site. Ringing commenced on 14th June 2013 during the experimental set up period. The bird feeders with RFID aerials collected data between 1st September 2013 (dashed line) and 30th August.

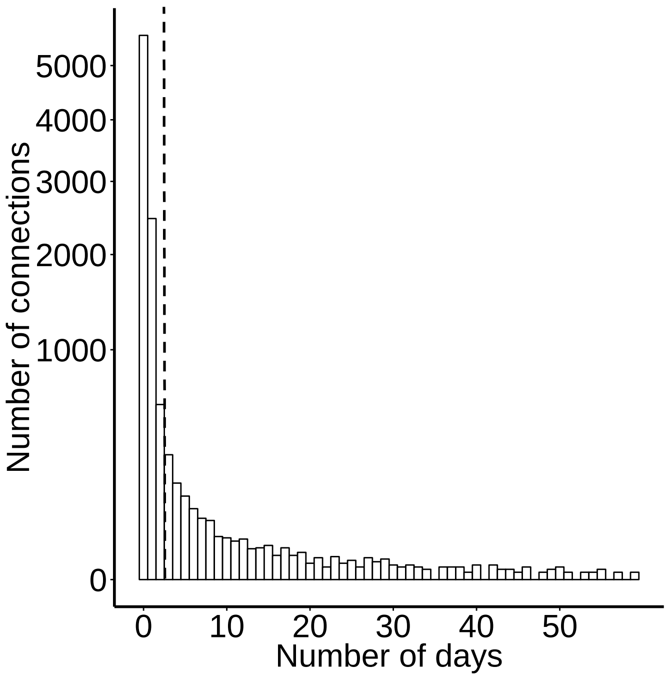

Figure S2. Time taken to form connections. Across all sites and seasons, a count of the number of days that tagged birds took to make a connection (i.e. to move between feeders in a pair). Left of the dashed line shows those birds used in the analysis (n = 8,652).

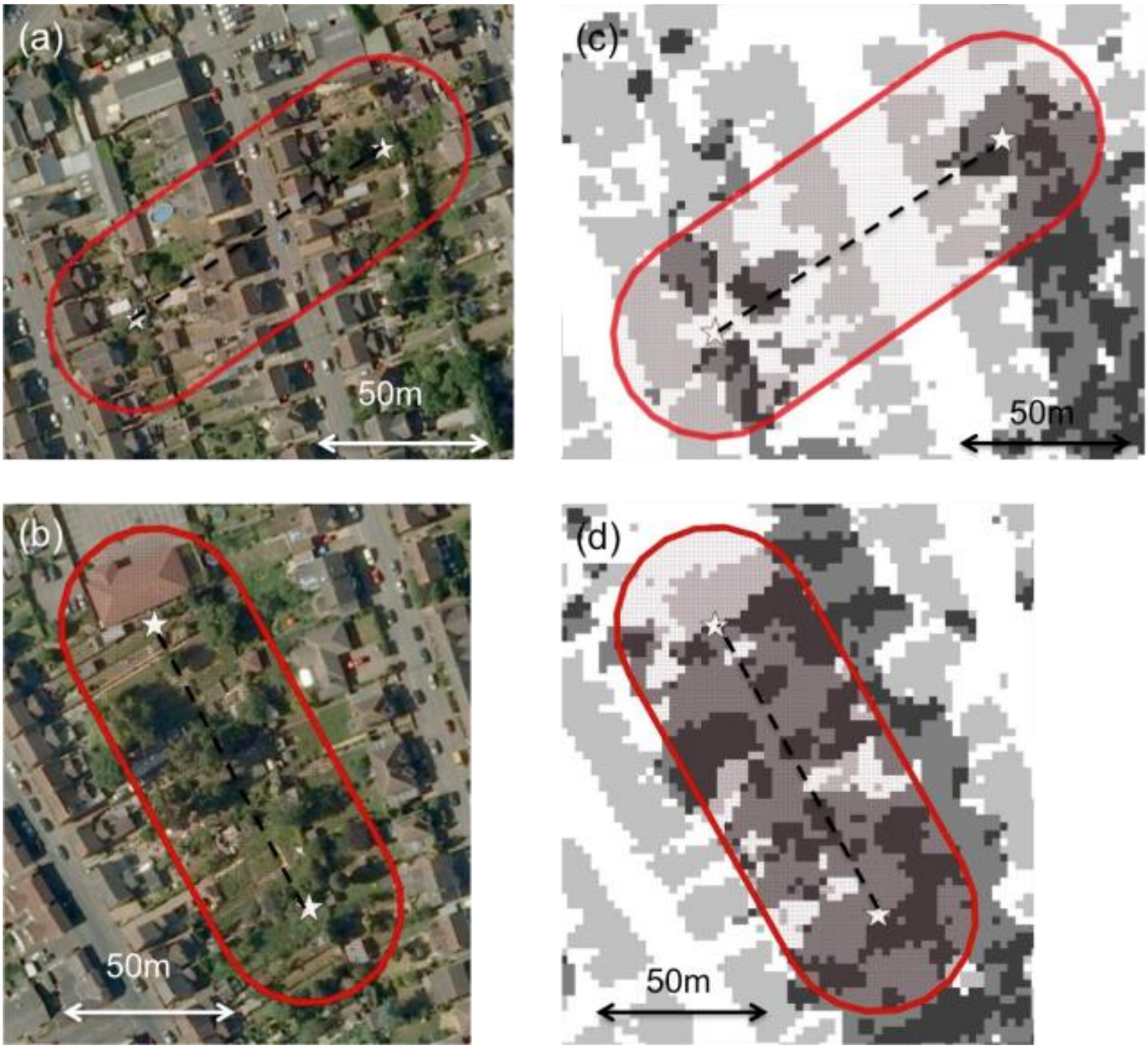

Figure S3. Examples of determination of habitat characteristics between feeder pairs; a) between green fragments, & b) within green fragments. The RFID bird feeders (★) are connected by a direct axis (dashed line). An ellipsoid was then created by applying a buffer (red line) around the axis. C-d) illustrates (a) & (b) using the hyper-spectral & LiDAR data used to calculated habitat classifications that were used in the analysis (white, vegetation free surfaces at ground level; light grey, buildings; medium grey, grass & low lying vegetation; dark grey, vegetation). Images a & b were sourced from Quantum GIS v2.6[5]; images c & d were generated using hyperspectral and LiDAR data (Appendix S1). The figure was created in Quantum GIS v2.6[5].

References

1 Tucker, C. J. Red and photographic infrared linear combinations for monitoring vegetation. *Remote Sens. Environ.* **8,** 127-150 (1979).

2 Liang, S. *Quantitative remote sensing of land surfaces*. (John Wiley & Sons, Inc., 2004).

3 Bradbury, R. B. *et al.* Modelling relationships between birds and vegetation structure using airborne LiDAR data: a review with case studies from agricultural and woodland environments. *Ibis* **147,** 443-452 (2005).

4 Hancock, S., Disney, M., Muller, J-P., Lewis, P. & Foster, M. A threshold insensitive method for locating the forest canopy top with waveform lidar. *Remote Sens. Environ.* **115,** 3286-3297 (2011).

5 Quantum GIS Development Team. Quantum GIS Geographic Information System v2.6. Open Source Geospatial Foundation Project. http://qgis.osgeo.org (2015).
